# Morphological Heterogeneity and Attachment of *Phaeobacter inhibens*

**DOI:** 10.1371/journal.pone.0141300

**Published:** 2015-11-11

**Authors:** Einat Segev, Adèle Tellez, Hera Vlamakis, Roberto Kolter

**Affiliations:** Department of Microbiology and Immunobiology, Harvard Medical School, Boston, Massachusetts, United States of America; Miami University, UNITED STATES

## Abstract

The Roseobacter clade is a key group of bacteria in the ocean exhibiting diverse metabolic repertoires and a wide range of symbiotic life-styles. Many Roseobacters possess remarkable capabilities of attachment to both biotic and abiotic surfaces. When attached to each other, these bacteria form multi-cellular structures called rosettes. *Phaeobacter inhibens*, a well-studied Roseobacter, exhibits various cell sizes and morphologies that are either associated with rosettes or occur as single cells. Here we describe the distribution of *P*. *inhibens* morphologies and rosettes within a population. We detect an N-acetylglucosamine-containing polysaccharide on the poles of some cells and at the center of all rosettes. We demonstrate that rosettes are formed by the attachment of individual cells at the polysaccharide-containing pole rather than by cell division. Finally, we show that *P*. *inhibens* attachment to abiotic surfaces is hindered by the presence of DNA from itself, but not from other bacteria. Taken together, our findings demonstrate that cell adhesiveness is likely to play a significant role in the life cycle of *P*. *inhibens* as well as other Roseobacters.

## Introduction

The Roseobacter clade is composed of heterotrophic alpha-proteobacteria that are highly abundant in the marine environment [1–3]. Roseobacters exhibit diverse metabolic capacities and are key players in many algae-bacteria interactions [2, 4–14].

Members of the Roseobacter clade are capable of rapidly colonizing various surfaces while outcompeting other bacteria [4, 13, 15, 16]. They can attach to both abiotic and biotic surfaces as well as to each other [4, 13, 15, 16]. When attached to each other via their poles, bacteria form multi-cellular structures in star-like shapes that are called rosettes. It has recently been shown that the attachment capability of *Phaeobacter inhibens*, a well-studied Roseobacter, is encoded by a plasmid [7]. Plasmid curing resulted in a mutant strain with severely perturbed adhesiveness [7]. Among other attachment deficiencies, the mutant strain was incapable of forming rosettes. Indeed, it has been previously suggested that rosette formation reflects the adhesive properties of Roseobacters [5].

Rosette formation is exhibited by many Roseobacter species and is affected by growth conditions, namely static cultures which promote rosette formation in comparison to shaking cultures [5]. Rosette structures have been documented in various alpha-proteobacteria [5, 17, 18]. While rosettes seem to be a morphological phenomenon distributed across the bacterial kingdom, the kinetics and mechanism of rosette formation are not well understood.

Another prominent morphological trait of several Roseobacters is the pleomorphic phenotype of cells in a population. In *Dinoroseobacter shibae* cells of various sizes and various modes of division have been reported [10]. A functional quorum sensing system is necessary to induce this physiological heterogeneity [10]. Phenotypic heterogeneity is emerging as a characteristic of various isogenic bacterial populations and has been proposed to serve as a survival strategy [19–22].

*P*. *inhibens* exhibits both the pleomorphic phenotype and the ability to form rosettes. Here, we describe the various morphologies of *P*. *inhibens* cells within a population. Through a combination of a fluorescent membrane staining and a fluorescently labeled lectin that stains a polar polysaccharide in *P*. *inhibens*, we distinguished between various cell types and determined the complexity of individual rosettes. Synchronization of the bacterial population allowed us to examine the morphology of cells and various properties of rosettes over time. By using lectins with different fluorescent labels, we then determined the kinetics of rosette formation. Finally, *P*. *inhibens* attachment to abiotic surfaces was examined using an attachment assay, allowing us to investigate how attachment capability changes over time and how it can be inhibited.

## Results

**Fig 1:**
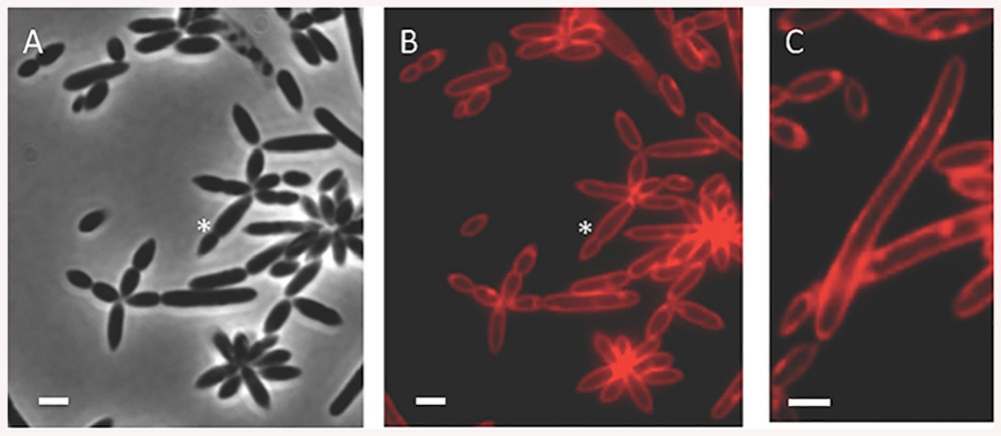
Morphological heterogeneity in *P*. *inhibens*. (A) Phase contrast image of cells from a mid-exponential phase culture. Asterisk indicates a pre-divisional constricted cell. (B) Fluorescent membrane staining of the cells shown in A. Asterisk indicates a septum. (C) Fluorescent membrane staining shows filamentous cells with no septa. Scale bars correspond to 1 μm.

To examine cell morphology in the bacterial population, we cultured *P*. *inhibens* bacteria to mid-exponential phase under nutrient rich growth conditions in 1/2YTSS medium, at 30°C shaking at 130 rpm. Samples of this culture were examined using phase contrast microscopy. Under these conditions, there was noticeable heterogeneity in the morphology of cells within the population (Fig 1A). We observed single cells as well as multicellular rosettes comprised of varying numbers of cells. Cell length in both rosettes and single cells varied considerably, ranging from around 1 μm up to 10 μm in length (Fig 1A). We considered cells greater than 3 μm to be filamentous and these long cells made up approximately 15% of the population. To determine whether filamentous cells are pre-divisional or continuous filaments, we used the fluorescent membrane stain, FM4-64 (see Methods). Examination of stained samples revealed no septa; we thus conclude that the filaments are long cells with one continuous cytoplasm (Fig 1B and 1C). Septa were observed in cells that also appeared to be constricted in the phase contrast image (for example, see cell adjacent to asterisk in Fig 1A and 1B). There did not appear to be a correlation between cell length and association with rosettes.

**Fig 2:**
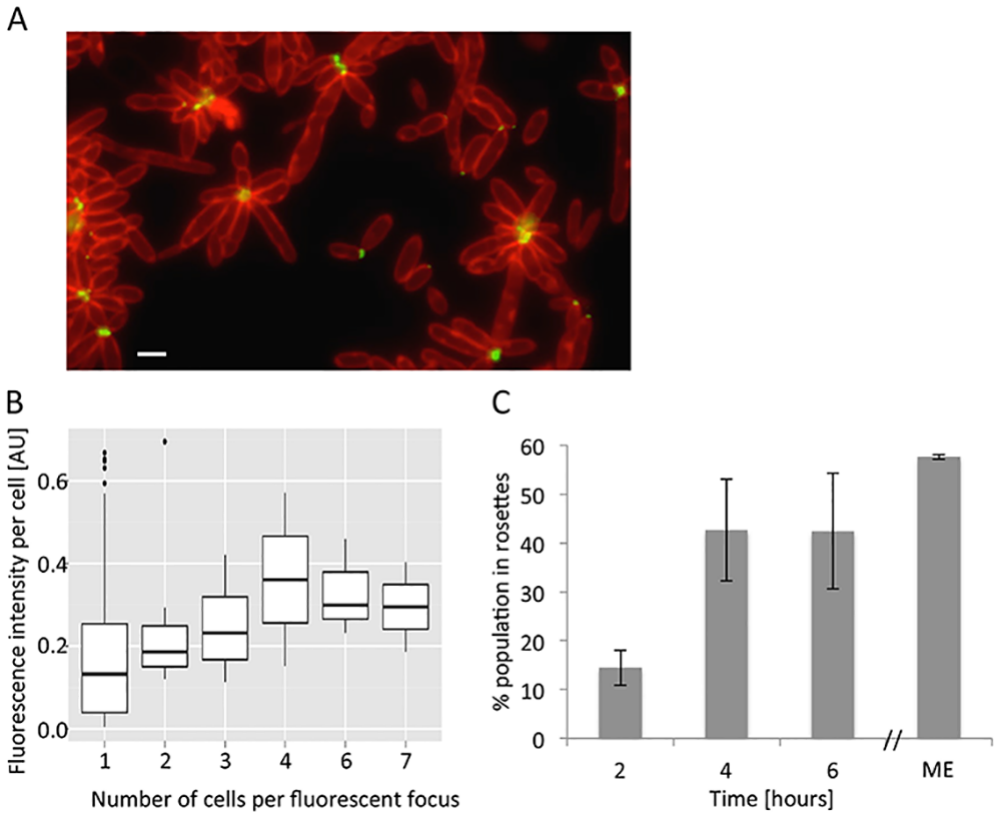
Polar polysaccharide at the center of rosettes. (A) Fluorescent overlay image of membrane stained cells (red) and Alexa 488-conjugated WGA lectin (green). Scale bar corresponds to 1 μm. (B) Quantification of lectin fluorescence intensity per cell in arbitrary units [AU]. Shown is a box plot generated with the R software [28]. (C) Percent of population in rosettes at early growth stages (2, 4, and 6 hours) or in a mid-exponential (ME) culture (see Methods). Cultures for this time course were obtained as described in Methods. Error bars indicate standard deviation of two biological replicates.

Cells in rosettes are attached through their pole to the rosette center. We hypothesized that *P*. *inhibens* might express a polar adhesive organelle. In other rosette-forming alpha-proteobacteria such as *Caulobacter crescentus* and *Agrobacterium tumefaciens*, polar attachment is mediated in part by a polysaccharide that can be detected with a Wheat Germ Agglutinin (WGA) lectin conjugated to a fluorophore [23–26]. Therefore, we stained cultures grown to mid-exponential phase with a fluorescently-conjugated WGA lectin, which preferentially binds to N-acetylglucosamine residues [25]. We detected bright fluorescent foci at one pole of a subset of the single cells and at the center of every rosette (Fig 2A). The foci observed in rosette centers appeared larger and brighter than the foci present in single cells. This made us wonder if cells within a rosette harbored more polar polysaccharides compared to individual cells (Fig 2A). To analyze the fluorescence intensity of the lectin-stained foci, we quantified the fluorescent signals using CellProfiler, an automated image analysis software for measuring biological phenotypes [27]. We divided the resulting values by the number of cells associated with every fluorescent focus thereby obtaining the fluorescent intensity per cell. These analyses indicated that fluorescence intensity per cell was not considerably increased if cells are associated with rosettes (Fig 2B). Thus, we conclude that all cells within a single rosette likely express similar amounts of a polar polysaccharide facing the rosette center. Attempts to stain our cultures with another lectin that specifically binds N-acetylgalactosamine (Dolichos Biflorus Agglutinin, vector laboratories) [26] did not yield any signal suggesting that this sugar is not part of the polar polysaccharide in *P*. *inhibens*.

**Fig 3:**
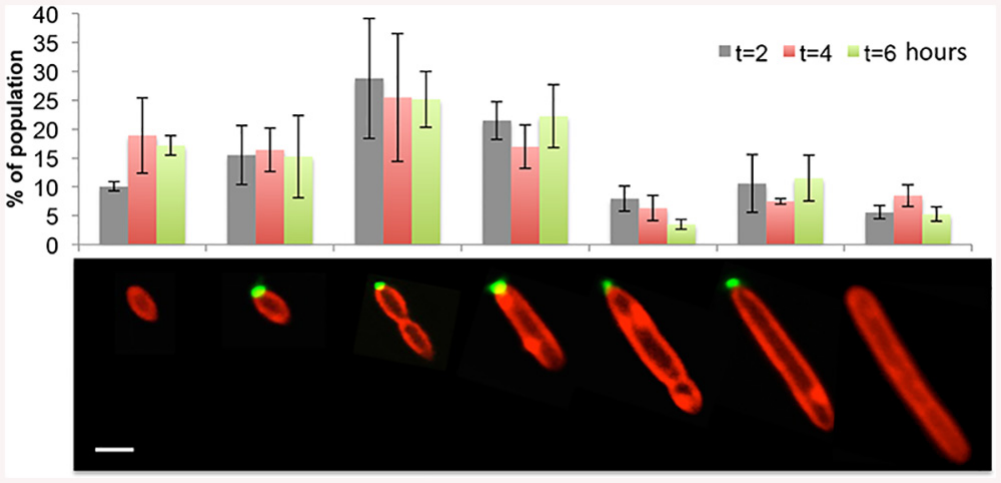
Cell type distribution does not vary with culture age. Seven different cell types were characterized by size, labeling with Alexa 488-conjugated WGA (green) and presence of a septum using membrane stain FM4-64 (red). Each cell type was quantified at 2, 4 and 6 hours of a culture in early growth stages; n > 300 cells. Scale bar corresponds to 1 μm.

In order to gain a better understanding of how rosettes are formed, we examined the very early stages of rosette formation starting with a relatively homogenous population. To this end, after cells were grown overnight shaking in flasks, we emptied the flasks and washed them several times with sterile water. Next, the flasks were filled with fresh 1/2YTSS medium and incubated at 30°C shaking at 130 rpm. Only cells that were strongly attached to the glass flask remained after the water washes and could further grow and divide once medium was added. Consequently, the starting population was synchronized and predominantly composed of single cells that were the progeny of the attached cells (S1A and S1B Fig). Since a biofilm forms overnight on the flask walls, we validated that our washing procedure eliminates any pre-formed biofilm that could serve as a source of cells later in the experiment (S1 Table). Of note, it is possible that cells or rosettes that were attached to the flask eventually detached and became part of the liquid culture. We then quantified the percent of the population that was in a rosette every two hours during the next six hours of growth. Given that under our experimental conditions the doubling time of *P*. *inhibens* is roughly 3 hours (S2 Fig), these time points provide information about the population prior to the first doubling, and after the second and third doublings. As the population grew, we observed an increase in rosette abundance between two and four hours post addition of fresh medium with approximately 45% of the cells participating in rosettes by four hours of growth (Fig 2C). After six hours there were still 45% of the cells in rosettes suggesting that the population may have reached equilibrium (Fig 2C). Indeed, when we quantified the number of cells in rosettes in the mid-exponential culture, we found that 57% of the cells were in rosettes, suggesting that the population did in fact reach a state in which approximately half of the cells participated in rosettes. Since cells within rosettes expressed a polar polysaccharide, we wanted to examine whether increased rosette abundance was due to increasing numbers of cells that express the polysaccharide over time. Therefore, we distinguished between seven different cell morphologies based on cell length, presence of a septum and presence of a polar polysaccharide (Fig 3). We then quantified the distribution of these cell morphologies in the entire population (single cells and rosettes) over time (Fig 3). Our results demonstrate a rather constant distribution of cell types over time (Fig 3). Thus, it does not seem that the increase in rosette abundance is driven by increasing numbers of lectin-stained cell types. Rather, it appears that the cells with polar polysaccharide are more likely to be in rosettes as the culture grows.

**Fig 4:**
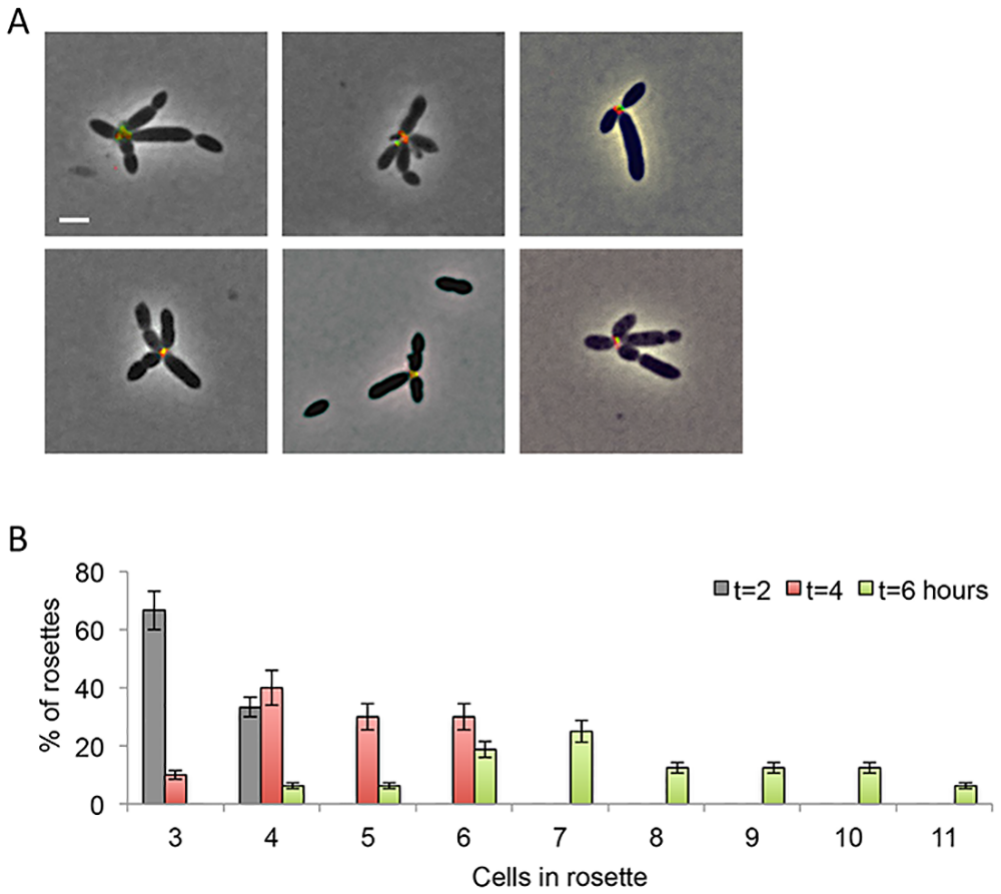
Kinetics of rosette formation. (A) Rosettes form by cell encounters. Two independent aliquots from the same synchronized culture were stained with either Alexa 488-conjugated WGA (green) or Alexa 594-conjugated WGA (red) and were mixed for 30 minutes at room temperature in buffer prior to imaging. Rosettes with dual-labeled centers are shown. Images are overlay of phase contrast (gray) with green and red fluorescence channels. (B) Rosette complexity increases over time. The number of cells per rosette was quantified over time. Error bars indicate standard deviation of two biological replicates; n > 300 cells.

There are two possible mechanisms by which the number of cells with polar polysaccharide would become enriched in rosettes: cells within rosettes divide and remain attached or cells expressing a polar polysaccharide encounter other cells and stick to each other. To distinguish between these possibilities, we first followed single cells through multiple cycles of division using time-lapse microscopy (S1, S2 and S3 Movies). We did not observe cells that divided into rosettes or that exhibited any sort of alignment towards a center. In order to investigate whether rosettes are capable of forming through bacterial encounter we devised a way to distinguish between bacteria within a rosette. We used two different fluorophores that fluoresce at different wavelengths (Alexa 488 and Alexa 594, Invitrogen) conjugated to the WGA lectin. For this experiment, a bacterial culture was synchronized (see Methods for details) and then split into two separate cultures. Each culture was stained with a different fluorophore and then thoroughly washed to discard any unbound lectin (see Methods). The two cultures were then mixed in buffer and incubated shaking at room temperature for 30 minutes. Importantly, these incubation conditions did not promote bacterial growth since we aimed to minimize cell division. Samples of the mixed culture were visualized under the microscope. As can be seen in Fig 4A, we detected rosettes in which both fluorophores were present in the rosette center.

In light of these results, we hypothesized that increasing cell numbers would raise the chance for bacterial encounter. This, in turn, could result in both increased rosette abundance (as was demonstrated in Fig 2C) and more cells per rosette over time. To test this hypothesis, we quantified the number of cells per rosette at two, four and six hours post inoculation. Indeed, over time rosettes exhibited increasing complexity with more cells per rosette (Fig 4B). At earlier time points (t = 2 in Fig 4B) very simple rosettes composed of 3–4 cells dominated the rosette fraction of the population. However at later time points (t = 6 in Fig 4B) rosettes that were composed of up to 11 cells could be seen. While we cannot rule out the possibility that cells might divide into rosettes, our results suggest that rosette formation is predominantly the result of cell encounters.

**Fig 5:**
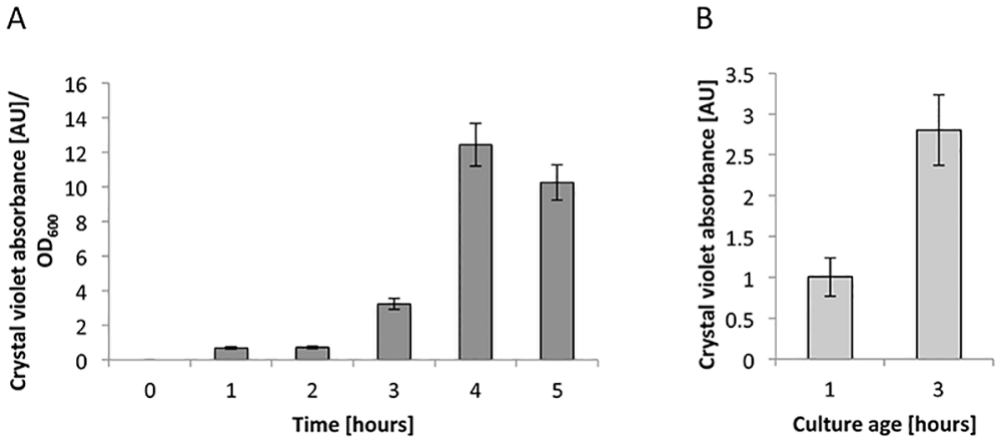
Attachment capability to an abiotic surface increases over time. (A) Ratio of crystal violet absorbance in arbitrary units over cell growth measured by OD_600_. (B) Cells of different ages were diluted to the same cell density prior to quantifying crystal violet absorbance of attached cells. Error bars indicate standard deviation of at least two biological replicates. Note: the 10-fold difference in crystal violet absorbance values between (A) and (B) is due to different incubation times during the attachment assay. See Methods for details.

In addition to attaching to each other to form rosettes, bacteria within the Roseobacter clade are well known for their ability to attach to abiotic surfaces. Therefore we wanted to examine whether increased rosette formation exhibited by *P*. *inhibens* over time, would be paralleled by an increased attachment capability to an abiotic surface. To assess bacterial attachment to an abiotic surface over time, we used an assay that was previously developed to assess biofilm formation [29], with slight modifications. Briefly, cells were grown over a period of five hours in multi-well plates. Every hour a plate was thoroughly washed with buffer and stained with crystal violet. To quantify the amount of attached biomass, the crystal violet stain was extracted with 33% acetic acid and absorbance of the solution at 595 nm was measured. Thus, absorbance measurements reflect the amount of cells attached to the well. Our analyses revealed that more cells attached to the well as the culture ages (Fig 5A). In this experiment, it was possible that the increased attachment was merely a result of more bacteria in the population and not that attachment capability increases over time. To address this, we wanted to examine similar numbers of bacterial cells in each time point, thereby eliminating differences in the population density. Therefore, we repeated the experiment using cells that were either from a one-hour culture or a three-hour culture. Samples were diluted to the same OD_600_ and the attachment assay was conducted 30 min post dilution. We observed that the cells originating from the three-hour culture had increased capability to attach (Fig 5B). Crystal violet staining revealed more than twice as much attachment in the three-hour culture compared to the one-hour culture (Fig 5B). Cells from these two time points demonstrate a comparable growth rate (S2 Fig) and thus differ only in their attachment capacity.

**Fig 6:**
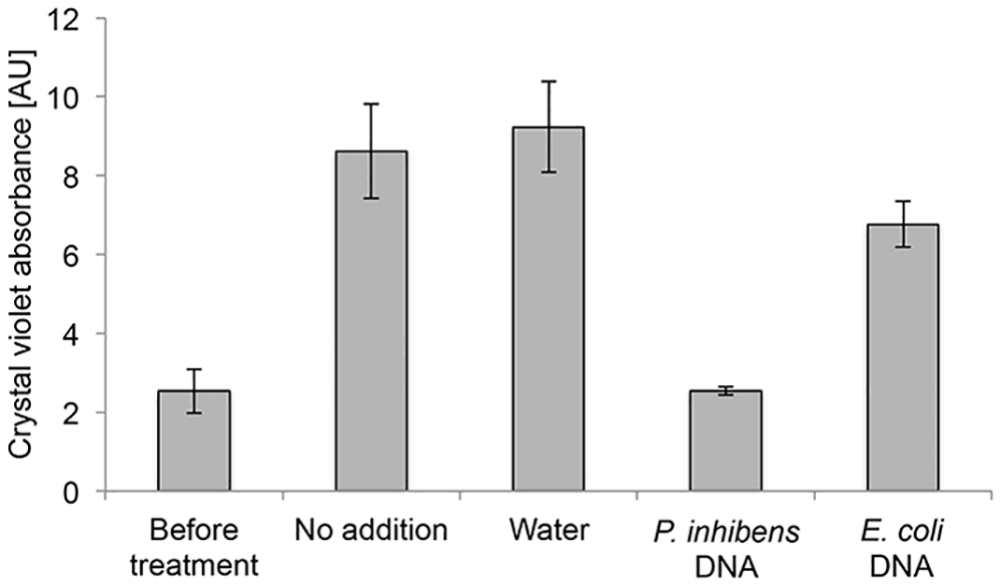
Self-DNA prevents attachment to an abiotic surface. Crystal violet absorbance of attached cells was quantified before and after addition of genomic DNA originating from either *P*. *inhibens* or *E*. *coli*. “Before treatment” measurement was conducted after 2 hours of incubation in an attachment assay (see Methods) and the different treatments were then applied. Measurements after treatments were conducted one hour following the addition of water or DNA. Error bars indicate standard deviation of at least two biological replicates. Note: crystal violet absorbance values before treatment and after treatment should be compared to time points 2 and 3 hours, respectively, in Fig 5A.

In *C*. *crescentus* the polar polysaccharide known as a holdfast is responsible for rosette formation and attachment to surfaces and is considered among the strongest natural adhesives with unique biophysical characteristics [25, 30, 31]. A unique feature of the *C*. *crescentus* holdfast is inhibition of attachment by DNA [32]. In this bacterium, DNA originating from *C*. *crescentus* cells (self-DNA) prevents attachment of *C*. *crescentus* cells to surfaces. However DNA from other bacteria does not have the same effect on *C*. *crescentus* cells [32]. In order to test whether attachment of *P*. *inhibens* was similarly affected by self-DNA, we carried out the attachment assay and after two hours, introduced either water or genomic DNA from the same strain of *P*. *inhibens* (Fig 6). Attachment was measured one hour after the addition of DNA. As can be seen in Fig 6, further attachment of bacteria was prevented following the addition of self-DNA. Of note, after two hours bacteria did further attach, however the amount of crystal violet staining was less during the assay in the sample where self-DNA was added (data not shown). These differences were statistically significant as determined using a Tukey multiple comparison post hoc test following a two-way ANOVA (see Methods for details). Since no measures were taken to prevent DNA degradation, it is possible that DNA was degraded over time resulting in less inhibition of attachment at later time points. To determine if the inhibition was specific, we added genomic DNA from *Escherichia coli*. Importantly, addition of foreign DNA that did not originate from *P*. *inhibens* bacteria did not have the same influence (Fig 6). Thus, we conclude that self-DNA perturbs the attachment capability of *P*. *inhibens*.

## Discussion

We have described the wide pleomorphism that *P*. *inhibens* cells display in nutrient rich medium with cell lengths ranging from one to ten microns. Under these conditions, approximately 50% of the population was found in rosettes whose complexity increased with time. Importantly, a subset of the *P*. *inhibens* cells express a polar polysaccharide that is present in all rosette centers. We found no evidence of rosette formation through cell division. Rather, we observed that rosettes could form through cell-cell encounters. Cell adhesiveness to other bacterial cells as well as to an abiotic surface increased over time. Moreover, the adhesiveness of cells to the abiotic surface was perturbed by self-DNA from *P*. *inhibens*, but not by DNA from *E*. *coli*. Interestingly, rosette formation was not inhibited by the addition of self-DNA (data not shown). Taken together, our results suggest that the polar polysaccharide plays a role in bacterial attachment but is likely to function in concert with additional cellular mechanisms.

The polar polysaccharide exhibited by *P*. *inhibens* shares several similarities with the well-studied holdfast of *C*. *crescentus*. In both organisms the polysaccharide localizes to one pole and contains N-acetylglucosamine residues [32]. In *C*. *crescentus* holdfast expression is associated with a distinct developmental stage, the transition from a motile to a sessile cell [33]. Holdfast expression is regulated throughout the bacterial cell cycle, resulting in optimal adhesive properties of *C*. *crescentus* during cell differentiation [34]. Whether *P*. *inhibens* possesses a similar cell cycle, and whether its polar polysaccharide is regulated in a similar manner, is yet to be determined.

In both *C*. *crescentus* and *P*. *inhibens* attachment to abiotic surfaces is inhibited by self-DNA [32]. In *C*. *crescentus* self-DNA adheres to the holdfast thereby preventing cells from attaching [32]. This inhibition mechanism precludes cells from irreversibly settling in a hostile environment where their siblings have lysed. It is likely that inhibition of attachment by self-DNA serves the same purpose in *P*. *inhibens*. Given that *P*. *inhibens* is so well adapted to attachment to surfaces and various eukaryotic hosts [4, 13, 15, 16], inhibitory signals such as self-DNA could aid cells in navigating towards desirable environments.

We found that the attachment of cells into rosettes appears to be the result of bacterial encounter. Whether rosettes are multi-cellular structures with designated functions or random clumps of sticky bacteria is yet to be determined. Various processes could be efficiently achieved through the formation of rosettes, *e*.*g*. DNA exchange through conjugation and buoyancy control. In the Gram-positive bacterium *Bacillus subtilis*, transfer of conjugative transposons is considerably more efficient when cells are in direct contact with each other within a chain [35]. Indeed, in the ocean, it would be beneficial to carry out horizontal gene transfer at a time when siblings are in close proximity. Thus rosettes might serve as a conjugational structure. Buoyancy control through multi-cellular structures is a well-studied phenomenon in yeasts where cells attach to each other in a process called flocculation and sink downwards [36]. Interestingly, attachment in flocculating yeasts is mediated by a specialized type of lectin-like molecules called zymolectins [36]. In the marine environment, a means to regulate vertical positioning through multi-cellularity could aid in following nutrient rich strata of water as well as avoiding strong irradiance in the sunlit surface waters.

It is not clear whether Roseobacters in the ocean live in rosettes. It has been reported that in environmental samples Roseobacters tend to fall into size fractions larger than free bacteria, and thus their abundance often is underestimated [37]. Whether the increased size reflects primarily association with particles (such as phytoplankton) or associations with each other in rosettes remains to be explored.

## Methods

### Strains and general growth conditions

The strain of *Phaeobacter inhibens* was DSM17395 purchased from the German collection of microorganisms and cell cultures (DSMZ, Braunschweig, Germany). Bacterial cultures were grown in liquid 1/2YTSS medium containing 2 g yeast extract (BD), 1.25 g tryptone (Sigma) and 20 g sea salt (Sigma) per liter. Cultures were incubated at 30°C shaking at 130 rpm.

#### Mid-exponential cultures

To examine the bacterial population at the mid-log phase, overnight cultures were diluted to OD_600_ = 0.05 in fresh liquid 1/2YTSS medium. Cultures were grown at 30°C shaking at 130 rpm. Cultures were examined after approximately 5 hours when they had reached an OD_600_ = 0.4.

#### Time course of synchronized cultures

To examine the early stages of rosette formation in the bacterial population, 10 ml cultures were grown overnight in 125 ml glass flasks. Flasks were then emptied, rinsed twice with sterile water, and filled with 10 ml of liquid 1/2YTSS medium, leaving only glass-adhered bacteria as the inoculum. Cultures were incubated at 30°C shaking at 130 rpm and were examined after 2, 4 and 6 hours.

#### Validating the absence of a biofilm prior to culture synchronization

A one Liter flask containing 50 ml of an overnight culture was rinsed twice with sterile water while a parallel one Liter flask containing 50 ml of an overnight culture was emptied but not rinsed. Both flasks were dried at 50°C for 20 min and then stained with 50 ml of 0.1% crystal violet for 15 min at room temperature. Following crystal violet staining, both flasks were thoroughly washed with 1x PBS and then filled with 50 ml 33% acetic acid. As a negative control, an empty flask was subjected to the same procedures. The absorbance of the extracted crystal violet was measured at 595 nm, values are shown in S1 Table.

### Light Microscopy

Fluorescence and phase contrast images were obtained using a Nikon TE-2000U inverted microscope equipped with a 100× Plan Apo NA 1.4 objective lens. All samples were spotted on thin 1X PBS with 1% agarose pads for visualization at room temperature. Images were acquired using a cooled Hamamatsu CCD camera controlled with MetaMorph 7 software (Molecular Devices). Alexa Fluor 488 conjugated WGA signals were captured using a narrow band eGFP filter (Chroma #41020). The membrane stain FM4-64 and the Alexa Fluor 594 conjugated WGA signals were visualized with the Chroma filter #62002v2. Images were analyzed using the MetaMorph 7 software. Images were processed identically for compared image sets.

#### Imaging of the synchronized cultures

A one Liter flask containing 50 ml of an overnight culture was thoroughly rinsed and then filled with 50 ml of fresh medium. The flask was incubated 20 min at 30°C shaking at 130 rpm. Next, the 50 ml were centrifuged at 8000 rpm for 30 min at 4°C. The pellet was stained with the fluorescent lectin as described below. Stained cells were imaged under the microscope as previously described.

#### Time lapse microscopy

To acquire movies of dividing cells, bacterial cultures were spotted on a square pad of agar-1/2YTSS. The pad was inverted onto a small petri-dish containing a glass bottom (MatTek). The microscope room was heated to 30°C prior to visualization. Phase contrast images were acquired as described above, every ten minutes for four hours. Movies were assembled using the MetaMorph 7 software.

### Fluorescent stains

#### Polar polysaccharide staining

Samples were incubated in the dark with Alexa Fluor 488 or 594 conjugated WGA (Life Technologies) for 30 min at room temperature. The stain final concentration was 5 μg/ml. Samples were rinsed twice in PBS prior to spotting on agarose pads for microscopy visualization.

#### Membrane staining

Samples were resuspended in 10 μg/ml FM4-64 immediately prior to visualization.

### Attachment assay

Our attachment assay is based on the previously developed assay for detection of biofilm attachment and formation [29] with the following modifications; overnight cultures were diluted to OD_600_ = 0.1 in fresh liquid 1/2YTSS medium. One ml of the diluted culture was placed per well in a 24-well plate and the plate was incubated at 30°C shaking at 130 rpm. A separate plate was prepared for each planned time point. At each indicated time point the wells were emptied and rinsed thoroughly with 1X PBS. Plates were then dried at 55°C for 20 min. After drying, wells were filled with 0.1% crystal violet and incubated at room temperature for 10 min. Wells were washed thoroughly with 1X PBS. Crystal violet was extracted using 33% acetic acid at room temperature for 15 min at 130 rpm. Absorption of crystal violet was measured using a spectrophotometer (Beckman DU 640) at wavelength of 595 nm.

#### Short attachment assay

For data shown in Fig 5B, the attachment assay was conducted as described above with the following modifications; an overnight culture was diluted to OD_600_ = 0.1 in fresh liquid 1/2YTSS medium and incubated at 30°C shaking at 130 rpm. Two hours later the same original over-night cultures was again diluted to OD_600_ = 0.1 in fresh liquid 1/2YTSS medium and incubated at 30°C shaking at 130 rpm. After an hour (when the culture that was diluted first completed three hours of incubation and the culture that was diluted second completed one hour of incubation) the older culture was diluted to the same OD_600_ as the younger culture. One ml of each diluted culture was placed in each well in a 24-well plate and the plates were incubated at 30°C shaking at 130 rpm. After 30 min the plates were processed as described above.

#### DNA addition

Total genomic DNA from *P*. *inhibens* or *Escherichia coli* was extracted using the wizard genomic DNA purification kit (Promega). DNA was added at the indicated time point at a final concentration of 50 μg/ml. The corresponding volume of water was added as control.

### Statistical analysis

To analyze statistical significance in Fig 6, two to three biological replicates per treatment, per time point were measured and analyzed with a two—way ANOVA (model statement: Absorbance ~ Treatment + Time + Treatment*Time). Both treatment and time factors were statistically significant, Treatment *P* < 0.0001, df = 3; Time *P* < 0.0001, df = 4, Treatment*Time *P* < 0.0001, df = 12.

## Supporting Information

S1 Fig Synchronizing the bacterial population. Twenty minutes after the introduction of fresh medium into the rinsed culture flask, the synchronized population is predominantly composed of small free-living cells that do not express a polar polysaccharide. (A) Overlay images of phase contrast microscopy and fluorescence of the fluorescently labeled lectin (see Methods for details). (B) Cell length histogram of the cells in the synchronized population. (C) Rosettes with the polar polysaccharide in their center could be detected, however they were low in frequency and included 22% of the cells in the population.
Scale bars correspond to 1 μm. n > 300 cells. Error bars indicate the standard deviation between two biological replicates.
(TIF)

S2 Fig *P*. *inhibens* growth curve. The growth of *P*. *inhibens* bacteria was monitored over 6 hours of growth. Error bars indicate the standard deviation between three biological replicates.
(TIF)

S1 Movie Time-lapse microscopy of a dividing *P*. *inhibens* cell. See Methods for details.
(MOV)

S2 Movie Time-lapse microscopy of a dividing *P*. *inhibens* cell. See Methods for details.
(MOV)

S3 Movie Time-lapse microscopy of a dividing *P*. *inhibens* cell. See Methods for details.
(MOV)

S1 Table Flask rinsing eliminates any pre-formed biofilm. Flasks containing overnight cultures were either rinsed (“Washed”) or emptied (“Unwashed”) and then stained with crystal violet (see Methods for details). A clean flask was subjected to the same procedure as a negative control. The measured crystal violet absorbance values demonstrate that the unwashed flask contains a significant biofilm that formed overnight, as exemplified by the high absorbance values. However the washed flask does not significantly differ in its absorbance values from the negative control. Error ranges indicate the standard deviation between two biological replicates (or technical replicates for the negative control).
(TIF)
