# Supplementary figures and images for: Morphological Heterogeneity and Attachment of Phaeobacter inhibens
Source: PLoS One. 2015 Nov 11;10(11):e0141300. doi: 10.1371/journal.pone.0141300 (PMC4641667; doi:10.1371/journal.pone.0141300)

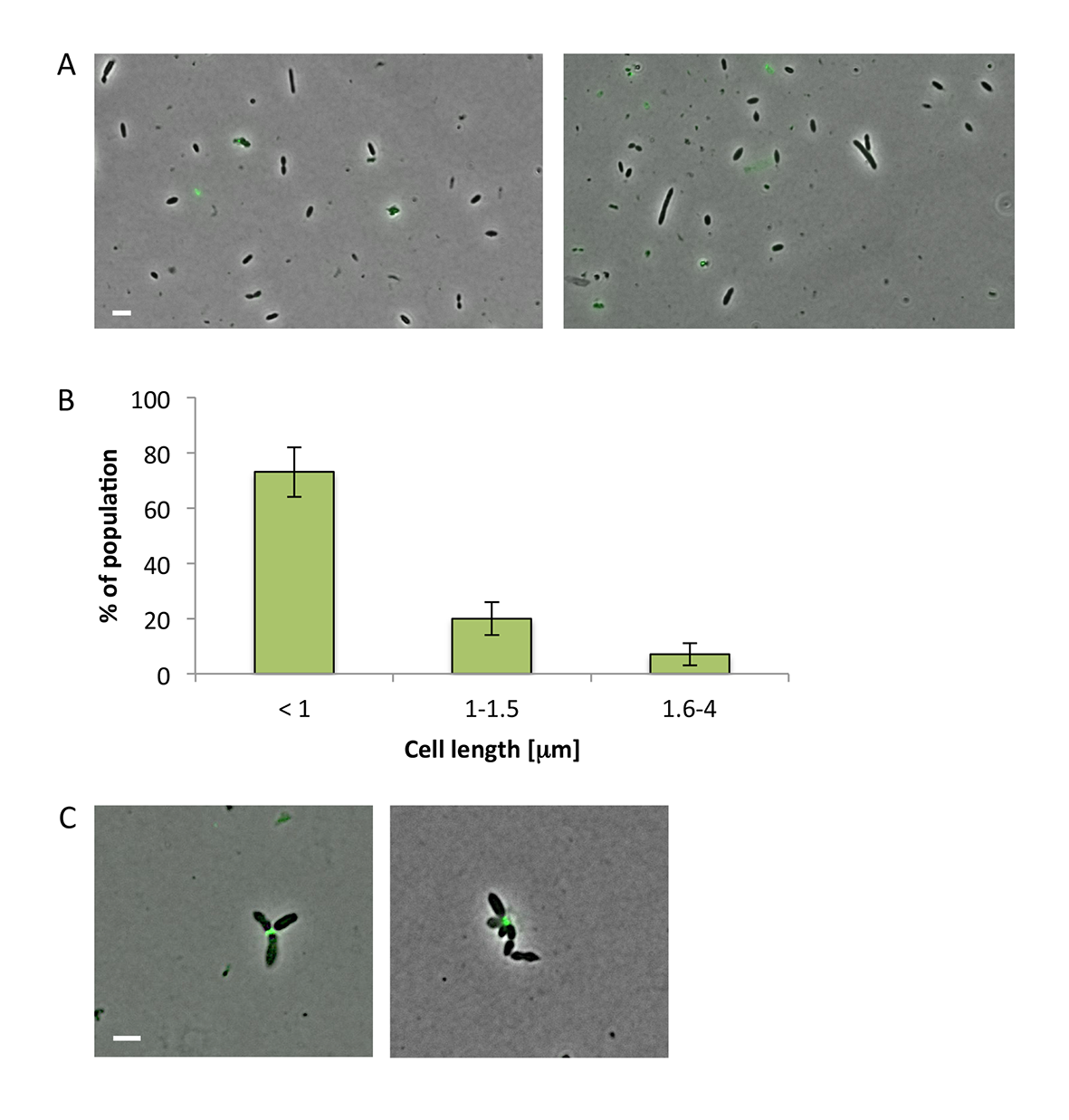

Supplement: S1 Fig — Twenty minutes after the introduction of fresh medium into the rinsed culture flask, the synchronized population is predominantly composed of small free-living cells that do not express a polar polysaccharide. (A) Overlay images of phase contrast microscopy and fluorescence of the fluorescently labeled lectin (see Methods for details). (B) Cell length histogram of the cells in the synchronized population. (C) Rosettes with the polar polysaccharide in their center could be detected, however they were low in frequency and included 22% of the cells in the population. Scale bars correspond to 1 μm. n > 300 cells. Error bars indicate the standard deviation between two biological replicates. (TIF) [file pone.0141300.s001.tif]

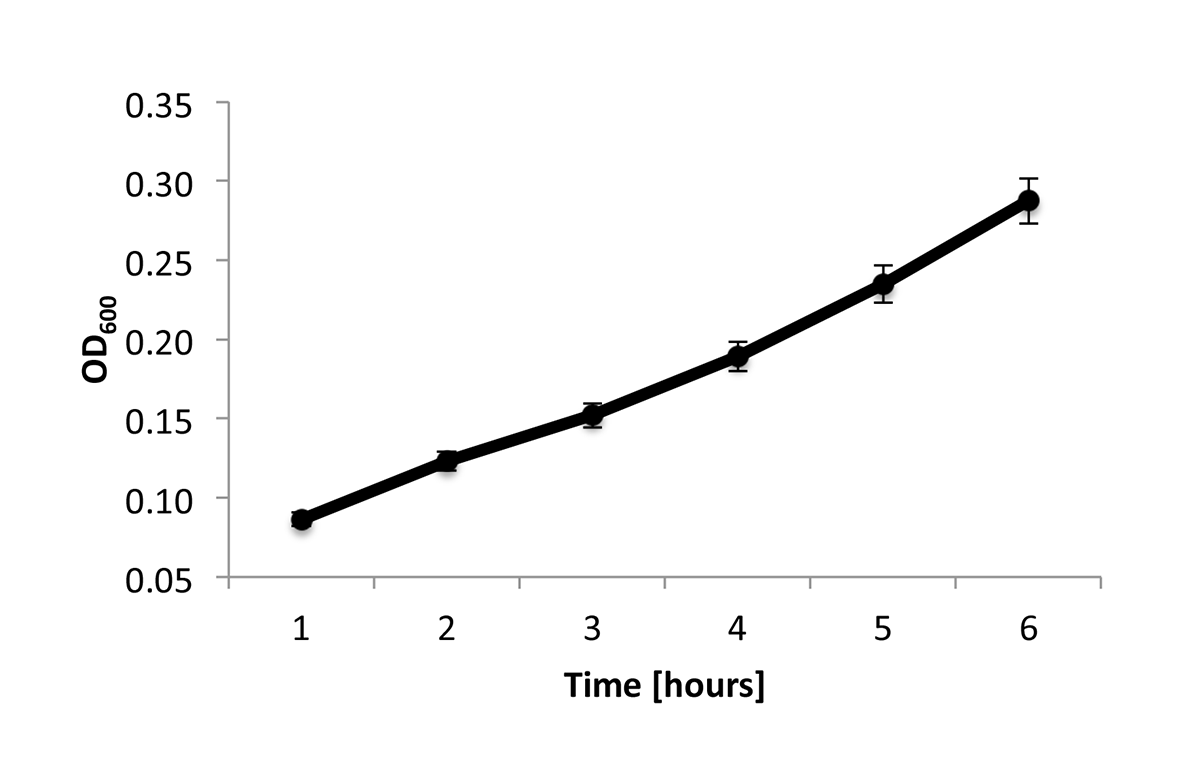

Supplement: S2 Fig — The growth of P. inhibens bacteria was monitored over 6 hours of growth. Error bars indicate the standard deviation between three biological replicates. (TIF) [file pone.0141300.s002.tif]

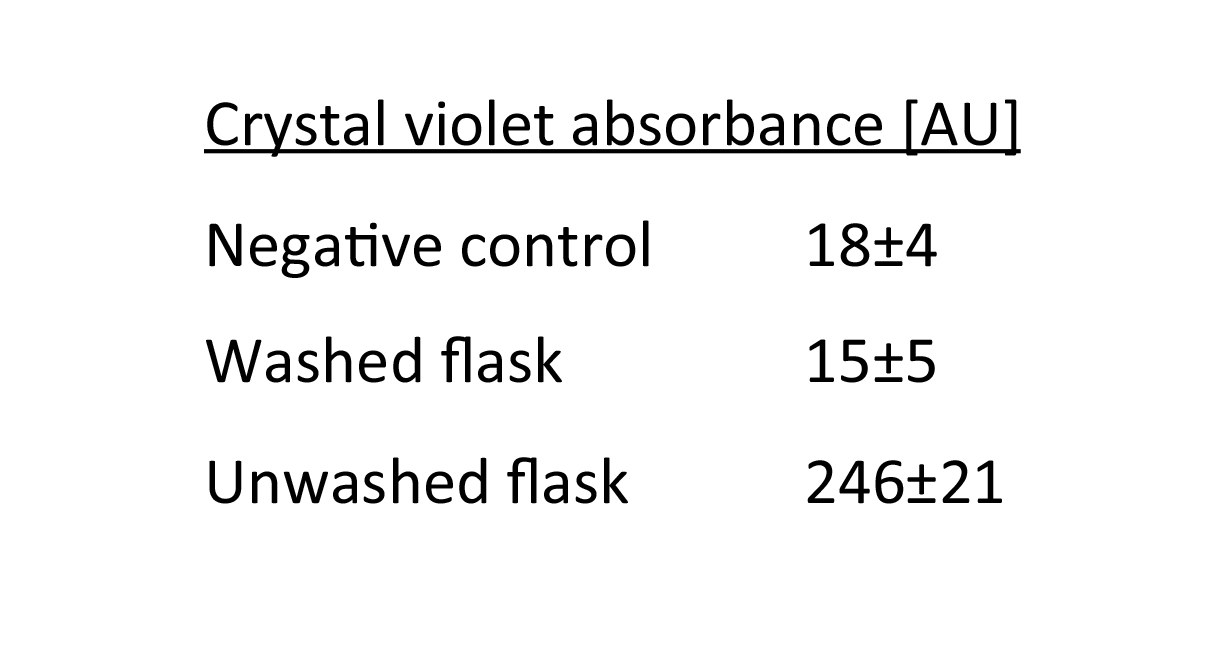

Supplement: S1 Table — Flasks containing overnight cultures were either rinsed (“Washed”) or emptied (“Unwashed”) and then stained with crystal violet (see Methods for details). A clean flask was subjected to the same procedure as a negative control. The measured crystal violet absorbance values demonstrate that the unwashed flask contains a significant biofilm that formed overnight, as exemplified by the high absorbance values. However the washed flask does not significantly differ in its absorbance values from the negative control. Error ranges indicate the standard deviation between two biological replicates (or technical replicates for the negative control). (TIF) [file pone.0141300.s006.tif]
